# Serologic Evidence of Influenza A(H1N1)pdm09 Virus Infection in Northern Sea Otters

**DOI:** 10.3201/eid2005.131890

**Published:** 2014-05

**Authors:** Zhu-Nan Li, Hon S. Ip, Jessica F. Trost, C. LeAnn White, Michael J. Murray, Paul J. Carney, Xiang-Jie Sun, James Stevens, Min Z. Levine, Jacqueline M. Katz

**Affiliations:** Centers for Disease Control and Prevention, Atlanta, Georgia, USA (Z.-N. Li, J.F. Trost, P.J. Carney, X.-J. Sun, J. Stevens, M.Z. Levine, J.M. Katz);; United States Geological Survey National Wildlife Health Center, Madison, Wisconsin, USA (H.S. Ip, C.L. White);; Monterey Bay Aquarium, Monterey, California, USA (M.J. Murray)

**Keywords:** influenza, sea otters, hemagglutination inhibition test, ELISA, influenza A(H1N1)pdm09, pandemic, viruses, respiratory infections, zoonoses, serology, northern sea otters, Enhydra lutris kenyoni, Washington, United States

**To the Editor:** Sporadic epizootics of pneumonia among marine mammals have been associated with multiple animal-origin influenza A virus subtypes (*1*–*6*); seals are the only known nonhuman host for influenza B viruses (*7*). Recently, we reported serologic evidence of influenza A virus infection in free-ranging northern sea otters (*Enhydra lutris kenyoni*) captured off the coast of Washington, USA, in August 2011 (*8*). To investigate further which influenza A virus subtype infected these otters, we tested serum samples from these otters by ELISA for antibody-binding activity against 12 recombinant hemagglutinins (rHAs) from 7 influenza A hemagglutinin (HA) subtypes and 2 lineages of influenza B virus (Technical Appendix Table 1). Estimated ages for the otters were 2–19 years (Technical Appendix Table 2); we also tested archived serum samples from sea otters of similar ages collected from a study conducted during 2001–2002 along the Washington coast (*9*). 

Of the 30 sea otter serum samples collected during 2011, a total of 21 (70%) had detectable IgG (>200) for rHA of influenza A(H1N1)pdm09 virus (pH1N1) strain A/Texas/05/2009. Four of 7 serum samples that showed IgG ≥6,400 against pH1N1 rHA also showed low cross-reactivity (IgG 200) against rHA of A/Brisbane/59/2007, a previous seasonal influenza A(H1N1) virus (Figure, panel A; Technical Appendix Table 1). No IgG was detected in any samples for any of the other 11 rHAs tested (IgG ≤100), and the sea otter serum samples collected during 2001–2002 did not react with any of the rHAs tested, including pH1N1 (IgG ≤100; Figure, panel A). 

**Figure:**
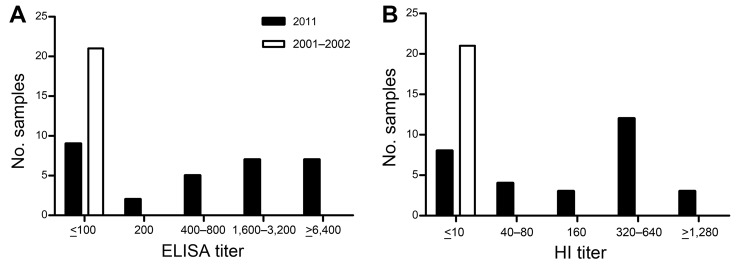
Results of ELISA and hemagglutination inhibition (HI) testing for influenza viruses in serum samples from northern sea otters captured off the coast of Washington, USA, during studies conducted in August 2011 (n = 30) and 2001–2002 (n = 21). A) IgG for influenza A(H1N1)pdm09 strain A/Texas/05/2009 detected by using standard indirect ELISA techniques with HRP-Protein A (Sigma, St. Louis, MO, USA). The ELISA titer was read as the reciprocal of the highest dilution of serum with an OD_450nm_ of >0.2 and 2-fold higher than the OD_450nm_ of control wells lacking serum. B) HI for influenza A(H1N1)pdm09 strain A/Mexico/4108/2009. HI titers were determined by using 0.5% turkey red blood cells (RBCs) for influenza A(H1N1)pdm09, seasonal influenza A(H1N1), influenza (H3N2), and influenza B viruses that circulated in North America during 2000–2011 and by using 1% horse RBCs supplemented with 0.5% BSA for avian influenza A(H1N1) virus strain A/duck/New York/96. HI assay was performed as described (www.who.int/influenza/gisrs_laboratory/manual_diagnosis_surveillance_influenza/en). OD, optical density.

Next, we tested serum samples by using a hemagglutination inhibition (HI) assay with whole influenza virus to detect strain-specific antibodies that inhibit receptor binding. Of the 30 samples collected during 2011, a total of 22 (73%) showed HI antibody titers of ≥40 against pH1N1 virus. Titers against all other human and avian viruses tested were ≤10 for all samples by HI assay using turkey red blood cells (RBCs) (Figure, panel B; Technical Appendix Table 3). No influenza A or B virus–specific HI antibodies were detected in the samples collected during 2001–2002 (data not shown). Although nasal swab specimens were collected from sea otters in the 2011 study, all specimens were negative for influenza virus by testing in embryonated eggs and by real-time PCR for detection of influenza A viral RNA (data not shown). These results suggest that sea otters were infected with influenza A virus sometime before the August 2011 sample collection date.

Although none of the 2011 samples showed HI titers to influenza A/duck/New York/96 (H1N1) virus (dk/NY/96) by testing using turkey RBCs (Technical Appendix Table 2), titers against this strain were detected when using horse RBCs, which is a more sensitive means for the detection of mammalian antibodies against some avian influenza subtypes (*10*). Of the 22 samples that had HI titers >40 to pH1N1 virus, 16 also had HI titers >40 against dk/NY/96 by horse RBC HI assay (Technical Appendix Table 2). However, titers against this strain were on average ≈4–8-fold lower than those for the pH1N1 virus strain, which suggests that that the titers against dk/NY/96 were the result of serologic cross-reactivity with avian- and swine-origin pH1N1 viruses. 

To further test for cross-reactivity, 4 sea otter serum samples were adsorbed with purified pH1N1 and dk/NY/96 virions. Adsorption with pH1N1, but not dk/NY/96, removed HI antibodies to pH1N1, whereas adsorption with either virus removed HI antibodies against dk/NY/96 (Technical Appendix Table 4). A comparison of amino acid sequences comprising the known HA antigenic sites on the pH1N1 structure confirmed high sequence identity and structural similarity with dk/NY/96 HA in Sa (12/13 aa residues) and Sb (8/12 aa residues) antigenic sites (data not shown). These results indicate that HI antibodies detected in sea otters are the result of pH1N1 virus infection but cross-react with the avian influenza A(H1N1) virus. 

Although we cannot exclude the possibility that sea otters were infected with classical swine influenza A(H1N1) virus, which shares high HA genetic and antigenic similarity with pH1N1 virus, our serologic evidence is consistent with isolation of pH1N1 virus from northern elephant seals (*1*). Therefore, we conclude that these sea otters were infected with pH1N1 virus. The origin and transmission route of pH1N1 virus infection in sea otters remain unknown. Potential contact between northern elephant seals and sea otters is one possibility; elephant seals’ summer feeding ranges and breeding areas along the Northeast Pacific coast overlap with areas where the Washington sea otter population is distributed (*1*).

In conclusion, our results show that sea otters are susceptible to infection with influenza A virus and highlight the complex nature of interspecies transmission of influenza viruses in the marine environment. Further surveillance, especially in other sea otter populations, is required to determine virus origin, potential pathogenesis, and consequences for the marine ecosystem.

Technical Appendix Titers for influenza A and B viruses detected in serum samples from northern sea otters captured off the coast of Washington, USA, during studies conducted in August 2011.
