# Supplementary material for: Serologic Evidence of Influenza A(H1N1)pdm09 Virus Infection in Northern Sea Otters
Source: Emerg Infect Dis. 2014 May;20(5):915–7. doi: 10.3201/eid2005.131890 (PMC4012822; doi:10.3201/eid2005.131890)
Supplement: Technical Appendix — Titers for influenza A and B viruses detected in serum samples from northern sea otters captured off the coast of Washington, USA, during studies conducted in August 2011. [file 13-1890-Techapp-s1.pdf]

Article DOI: http://dx.doi.org/10.3201/eid2005.131890

Serologic Evidence of Influenza A(H1N1)pdm09 Virus in Northern Sea Otters

Technical Appendix

Technical Appendix Table 1. HA specific IgG ELISA titer range detected in sea otter sera collected in 2011

	Number of sera with ELISA titer range				
rHA	≤100	200	400–800	1600–3200	≥6400
A/Texas/05/09 [A(H1N1)pdm09]	9	2	5	7	7
A/Brisbane/59/07 (H1N1)	26	4	0	0	0
A/Japan/305/57 (H2N2)	30	0	0	0	0
A/Hong Kong/1/68 (H3N2)	30	0	0	0	0
A/swine/Wisconsin/12/10 (H3N2v)	30	0	0	0	0
A/Wisconsin/67/05 (H3N2)	30	0	0	0	0
A/Vietnam/1203/04 (H5N1)	30	0	0	0	0
A/Netherlands/219/03 (H7N7)	30	0	0	0	0
A/Hong Kong/1073/99 (H9N2)	30	0	0	0	0
A/shorebird/Delaware/68/04 (H13N9)	30	0	0	0	0
B/Brisbane/60/08	30	0	0	0	0
B/Wisconsin/01/10	30	0	0	0	0

Technical Appendix Table 2. HI titers of sea otter sera collected in 2011 against A/Mexico/4108/09 [A(H1N1)pdm09] and A/duck/NY/1996 viruses

		A/Mexico/4108/09		A/duck/New York/96	
Sample	Age	tRBC HI	hRBC HI	tRBC HI	hRBC HI
1000	2	640	ND*	10	80
1001	6	320	ND	10	40
1002	7	320	ND	10	80
1003	8	1280	ND	10	160
1004	7	80	ND	10	10
1005	12	5	ND	10	10
1006	5	640	ND	10	160
1007	19	160	ND	10	160
1008	5	1280	ND	10	160
1009	5	80	ND	10	10
1010	10	5	ND	10	10
1011	3	160	ND	10	10
1012	9	640	ND	10	160
1013	6	5	ND	10	10
1014	10	80	ND	10	10
1015	7	5	ND	10	10
1016	6	320	ND	10	160
1017	10	5	ND	10	10
1018	10	40	ND	10	10
1019	11	320	ND	10	80
1020	7	5	ND	10	10
1021	7	160	ND	10	20
1022	6	640	ND	10	160
1023	6	320	ND	10	80
1024	12	5	ND	10	10
1025	4	640	ND	10	80
1026	13	5	ND	10	10
1027	6	640	ND	10	320
1028	3	640	ND	10	80
1029	7	1280	ND	10	160

*ND, not done. Horse red blood cell HAU<4 for A/Mexico/4108/09 virus

Technical Appendix Table 3. HI titer range against influenza A and B viruses detected in sea otter sera collected in 2011

Virus	Number of sera with HI titer range				
	≤10	40–80	160	320–640	≥1280
A/Mexico/4108/09 [A(H1N1)pdm09]	8	4	3	12	3
A/New Caledonia/20/99 (sH1N1)	30	0	0	0	0
A/Brisbane/59/07 (sH1N1)	30	0	0	0	0
A/duck/New York/96 (aH1N1)	30	0	0	0	0
A/Panama/2007/99 (H3N2)	30	0	0	0	0
A/Perth/16/09 (H3N2)	30	0	0	0	0
B/Beijing/184/93	30	0	0	0	0
B/Florida/4/06	30	0	0	0	0
B/Brisbane/60/08	30	0	0	0	0

Technical Appendix Table 4. Sea otter HI titers against A/Mexico/4108/09 [A(H1N1)pdm09] and A/duck/New York/96 viruses after serum adsorption with A(H1N1)pdm09 and A/duck/NY/96 virions.

Serum sample	HI titer to A(H1N1)pdm09* after adsorption with			HI titer to A/duck/NY/96† after adsorption with		
	Mock	A(H1N1)pdm09	A/duck/New York/96	Mock	A(H1N1)pdm09	A/duck/New York/96
1001	80	5	80	40	5	5
1007	80	5	40	80	5	5
1016	320	5	320	80	5	5
1019	160	5	160	40	5	5

*0.5% turkey RBCs were used
†1% horse RBCs supplemented with 0.5% BSA were used
